# Bladder cancer during the COVID-19 pandemic: the calm before the storm?

**DOI:** 10.2144/fsoa-2020-0101

**Published:** 2020-08-03

**Authors:** Julien Sarkis, Ramy Samaha, Joseph Kattan, Pierre Sarkis

**Affiliations:** 1 Department of Urology, Hotel-Dieu de France Hospital, Beirut, Lebanon; 2 Department of Hematology & Oncology, Hotel-Dieu de France Hospital, Beirut, Lebanon; 3 Department of Urology, Saint Joseph Hospital, Beirut, Lebanon

**Keywords:** BCG therapy, bladder cancer, coronavirus, COVID-19, pembrolizumab

A rise in bladder cancer deaths is expected in the post-COVID-19 era and practical solutions must be rapidly found to delay what would possibly be the real ‘second wave’ of deaths due to this pandemic

First described in December 2019, the novel coronavirus (2019-nCoV or COVID-19) has been ravaging the world since [1]. Since the start of the outbreak, most countries have been facing a major public health crisis: declared as a pandemic by the WHO (Geneva, Switzerland), SARS-CoV-2 has already registered more than 4 million cases and around 300,000 deaths worldwide [2]. While the most common clinical manifestations are nonspecific (fever, cough, fatigue and shortness of breath), pneumonia and acute respiratory distress syndrome [3] can occur and are generally life threatening, especially in the immunocompromised population [4]. Thus, cancer patients are considered a high-risk population: their cancer related treatments, as well as cancer itself result in an immunosuppressive state rendering them highly susceptible to infectious complications [5]. A Chinese nationwide analysis conducted by Liang *et al.* effectively concluded that cancer patients had a higher risk of severe events that required admission to the intensive care unit (ICU), ventilation support or that resulted in death [6,7]. In order to protect their patients, the oncology community swiftly responded and developed interim guidelines and practice changes to get through this pandemic [8,9]. Some of these measures include delay or omission of treatment, delayed surgery utilizing neoadjuvant chemotherapy as a bridge and choosing marginally less effective treatments but with lower risk of immunosuppression. While these measures aim at reducing contagion, they cause a disruption to the spectrum of medical cancer care services [10], with a potential negative effect on cancer detection and prognosis. A rise in cancer incidence, a shift toward more advanced stage disease and a higher mortality rates can be expected in the coming years. In this paper, we highlight the particular case of bladder cancer (BC) as well as the numerous factors that can cause a peak in BC cases in the post-COVID-19 era. We also propose different solutions to avoid a future oncologic crisis.

## COVID-19 repercussions on bladder cancer

Patient’s fear, associated to the difficulty in engaging with healthcare system, are the main factors that are leading to delays in the diagnostic algorithm and a subsequent progression of undetected bladder cancers. Indeed, bladder cancer occurs mainly in the elderly population with an average age at the time of diagnosis of 73 [11]. However, worries about contracting COVID-19 and fear of its presence in healthcare facilities have discouraged this category of patients from contacting their doctors, as up to 80% of deaths due to COVID-19 occurred among adults aged ≥60 years [12]. Bladder cancer symptoms like macroscopic hematuria can be therefore minimized and consequently overlooked. This is added to the fact that most centers have reduced their outpatient capacity and are doing a triage to only select patients that are in most need of a cystoscopy [13].

Even patients already diagnosed with bladder cancer can see their treatment (transurethral resection of bladder tumor, intravesical instillations or radical cystectomy) delayed. Transurethral resection of bladder tumor, the cornerstone management of nonmuscle invasive bladder cancer (NMIBC), has no current alternative [14]. However, during this critical period during which hospital beds must be released to accommodate the influx of COVID-19 positive patients, admissions to the urology, oncology or radiotherapy departments are being avoided [13]. Consequently, cancer committees have drawn up recommendations to distinguish NMIBC requiring urgent treatment from those whose treatment can be deferred [15]. Validated clinical and pathological criteria (previous history, tumor size and number) can help physicians in evaluating which tumors necessitate urgent resection from those that can be deferred. But even in low-risk cases, resection cannot be postponed for more than 3 months [15]. Patients may therefore find it difficult to meet this timeline and cancer progression may be observed. This problem is even more encountered in high-risk NMIBC patients as they require regular hospital visits for intravesical therapy. The latter, like Bacillus Calmette–Guerin (BCG) instillations, cannot be reduced [16] despite the current crisis. And finally, due to operating room closure and saturation of ICU beds, muscle-invasive bladder cancer patients awaiting their radical cystectomy are seeing their surgery delayed [9]. This is problematic, especially for patients on neoadjuvant chemotherapy as a delay of more than 3 months may have a negative impact on prognosis [17]. Some muscle-invasive bladder cancer patients may even undergo suboptimal treatment like radiotherapy and chemotherapy instead of surgery.

## Flattening the curve

Solutions must be rapidly identified as delay in BC management will cause fatal progression of the disease and presentation at a more advanced stage, which will eventually require urgent hospital admission. Patients should know that the risks of delayed emergency conditions due to BC can be much higher than those posed by COVID-19. Measures that raise patient awareness and allow care at home like telemedicine and telephone calls should be encouraged [18]. Patient’s follow-up must be preferably performed by teleconsultation to avoid their displacements and in case of BC suspicion, physicians must be able to assure primary diagnostic tests like urine cytology, urine culture and ultrasonography in COVID-19 free centers to ensure patient’s safety. A safe environment should also be insured for BC patients under instillation therapy (BCG or chemotherapy), as their regular hospital visits may expose them to the virus. On another note, because complex cases and life-saving surgeries are being postponed, it is essential to maintain traceable multidisciplinary discussions [19]. The latter can also propose evidence-based alternatives for surgeries, without compromising prognosis. Recently US FDA approved pembrolizumab, for example, can be discussed in BCG-unresponsive, high-risk, NMIBC with carcinoma *in situ* that cannot undergo cystectomy (Phase II KEYNOTE-057 trial [20]) because of closed operating rooms or saturated ICU beds.

## Conclusion & future perspective

The anticipated rise in bladder cancer in the following months is another face of the global public health impact of COVID-19. Delayed cancer diagnoses and management during the pandemic, risks many thousands of BC cases going undetected and untreated, causing a surge in untreated BC incidence in the coming months. This surge in advanced bladder cancer, as well as the knock-on effects (rise in demand for cancer-related services once the pandemic has passed), “*could overwhelm health services and contribute to an excess in cancer-related mortality in the coming years”’* [10]. A practical proactive approach must be started now, more than ever to delay what would possibly be the real ‘second wave’ of deaths due to COVID-19.
